# Retraction: Impact of a Child Sexual Abuse Prevention Package (CSAPP) on Knowledge of Appropriate and Inappropriate Touch Among Children With Intellectual Disabilities

**DOI:** 10.7759/cureus.r252

**Published:** 2026-10-09

**Authors:** Ayantika Biswas, Hepsi Bai Joseph, Asha Shetty

**Affiliations:** 1 College of Nursing, All India Institute of Medical Sciences, Bhubaneswar, Bhubaneswar, IND

This article has been retracted by the Editor-in-Chief due to concerns regarding the informed consent process for the study participants, who were minors with intellectual disabilities. The authors disagree with the decision to retract.

